# Some New Sets of Sequences of Fuzzy Numbers with Respect to the Partial Metric

**DOI:** 10.1155/2015/735703

**Published:** 2015-01-28

**Authors:** Uğur Kadak, Muharrem Ozluk

**Affiliations:** ^1^Department of Mathematics, Faculty of Sciences, Gazi University, 06500 Ankara, Turkey; ^2^Department of Mathematics, Faculty of Sciences and Arts, Bozok University, 66100 Yozgat, Turkey; ^3^Department of Mathematics, Faculty of Sciences and Arts, Batman University, 72060 Batman, Turkey

## Abstract

In this paper, we essentially deal with Köthe-Toeplitz duals of fuzzy level sets defined using a partial metric. Since the utilization of Zadeh's extension principle is quite difficult in practice, we prefer the idea of level sets in order to construct some classical notions. In this paper, we present the sets of bounded, convergent, and null series and the set of sequences of bounded variation of fuzzy level sets, based on the partial metric. We examine the relationships between these sets and their classical forms and give some properties including definitions, propositions, and various kinds of partial metric spaces of fuzzy level sets. Furthermore, we study some of their properties like completeness and duality. Finally, we obtain the Köthe-Toeplitz duals of fuzzy level sets with respect to the partial metric based on a partial ordering.

## 1. Introduction

By *ω*(*F*), we denote the set of all sequences of fuzzy numbers. We define the classical sets *bs*(*H*), *cs*(*H*), and *cs*_0_(*H*) consisting of the sets of all bounded, convergent, and null series, respectively; that is

(1) bsH:=u=uk∈ωF:∑k=0nuk∈l∞H,csH:=u=uk∈ωF:∑k=0nuk∈cH,cs0(H):=u=uk∈ωF:∑k=0nuk∈c0H.

We can show that *bs*(*H*), *cs*(*H*), and *cs*_0_(*H*) are complete metric spaces with the partial metric *H* ^*s*^ defined by

(2) $$\begin{array}{c} H_{\infty }^{p}\left(u,v\right):=\underset{n\in N}{\sup}\left\{\overset{n}{\underset{k=0}{\sum }}H^{s}\left(u_{k},v_{k}\right)\right\}, \end{array}$$

where *u* = (*u*_*k*_) and *v* = (*v*_*k*_) are the elements of the sets *bs*(*H*), *cs*(*H*), or *cs*_0_(*H*).

Secondly, we introduce the sets *bv*(*H*), *bv*_*q*_(*H*), and *bv*_*∞*_(*H*) consisting of sequences of *q*-bounded variation by using the partial metric *H* ^*s*^ with respect to the partial ordering ⊑_*H*_, as follows:

(3) lbvH:=u=uk∈ωF:∑k=0∞Hs[Δuk,0¯]<∞,bvqH:=u=uk∈ωF:∑k=0∞HsΔuk,0¯q<∞,bv∞H:=u=uk∈ωF:sup⁡k∈NHsΔuk,0¯<∞,

where the distance function *H* ^*s*^ denotes the partial metric of fuzzy level sets defined by

(4) Hu,v=sup⁡λ∈0,1p(uλ,vλ)   l=sup⁡λ∈[0,1]max⁡{uλ+,vλ+}−min⁡⁡uλ−,vλ− kkkkl=max⁡u0+,v0+−min⁡u0−,v0−,Hsu,v=2Hu,v−Hu,u−Hv,v,

for any *u*, *v* ∈ *E* ^1^ with the partial ordering ⊑_*H*_. One can conclude that the sets *bv*(*H*), *bv*_*q*_(*H*), and *bv*_*∞*_(*H*) are complete metric spaces with the following partial metrics:

(5) HΔ(u,v):=∑k=0∞Hs[Δuk,Δvk],HqΔu,v:=∑k=0∞HsΔuk,Δvkq1/q,H∞Δ(u,v):=sup⁡k∈N Hs[Δuk,Δvk],

respectively, where *u* = (*u*_*k*_) and *v* = (*v*_*k*_) are the elements of the sets *bv*(*H*), *bv*_*q*_(*H*), or *bv*_*∞*_(*H*) and (Δ*u*)_*k*_ = *u*_*k*_ − *u*_*k*+1_ for all *k* ∈ *N*.

Many authors have extensively developed the theory of the different sets of sequences and its matrix transformations [1, 2]. Following Başar [3, page 347], we note that Mursaleen and Basarır [4] have recently introduced some new sets of sequences of fuzzy numbers generated by a nonnegative regular matrix *A* some of which reduced to the Maddox's spaces *l*_*∞*_(*p*; *F*), *c*(*p*; *F*), *c*_0_(*p*; *F*), and *l*(*p*; *F*) of sequences of fuzzy numbers for the special cases of that matrix *A*. Quite recently, Talo and Başar [5] have extended the main results of Başar and Altay [6] to fuzzy numbers and defined the alpha-, beta-, and gamma-duals of a set of sequences of fuzzy numbers and gave the duals of the classical sets of sequences of fuzzy numbers together with the characterization of the classes of infinite matrices of fuzzy numbers transforming one of the classical set into another one. Also, Kadak and Başar [7–9] have recently studied fourier series of fuzzy valued functions and gave some properties of the level sets together with some inclusion relations, in [10]. Finally, Kadak and Ozluk [11–13] have introduced the sets *l*_*∞*_(*H*), *c*(*H*), *c*_0_(*H*), and *l*_*p*_(*H*) of classical sequences of fuzzy level sets and sufficient conditions for partial completeness of these are established by means of fuzzy level sets.

The rest of this paper is organized as follows. In Section 2, some required definitions and consequences related with the partial metric and fuzzy level sets, sequences, and convergence are given.  Section 3 is devoted to the completeness of the sets of sequences *bs*(*H*), *cs*(*H*), *cs*_0_(*H*) and *bv*(*H*), *bv*_*∞*_(*H*), *bv*_*q*_(*H*) of fuzzy level sets and some related notions. In the final section of the paper, the Köthe-Toeplitz duals of some classical sets are determined and given some properties including solidness.

## 2. Preliminaries, Background and Notation

Motivated by experience from computer science, nonzero self-distance seen to be plausible for the subject of finite and infinite sequences.

**Definition 1 (see [14]).**

Let *X* be a nonempty set and *p* be a function from *X* × *X* to the set *R* ^+^ of nonnegative real numbers. Then the pair (*X*, *p*) is called a* partial metric space* and *p* is a* partial metric* for *X*, if the following partial metric axioms are satisfied for all *x*, *y*, *z* ∈ *X*:

(P1) *x* = *y* if and only if *p*(*x*, *x*) = *p*(*x*, *y*) = *p*(*y*, *y*),
(P2) 0 ≤ *p*(*x*, *x*) ≤ *p*(*x*, *y*),
(P3) *p*(*x*, *y*) = *p*(*y*, *x*),
(P4) *p*(*x*, *z*) ≤ *p*(*x*, *y*) + *p*(*y*, *z*) − *p*(*y*, *y*).

**Proposition 2 (Nonzero self-distance [15]).**

Let *S* ^*ω*^ be the set of all infinite sequences *x* = (*x*_0_, *x*_1_, *x*_2_,…) over a set *S*. For all such sequences *x* and *y* let *d*_*s*_(*x*, *y*) = 2^−*k*^, where *k* is the largest number (possibly *∞*) such that *x*_*i*_ = *y*_*i*_ for each *i* < *k*. Thus *d*_*s*_(*x*, *y*) is defined to be 1 over 2 to the power of the length of the longest initial sequence common to both *x* and *y*. It can be shown that (*S* ^*ω*^, *d*_*s*_) is a metric space.

Each partial metric space thus gives rise to a metric space with the additional notion of nonzero self-distance introduced. Also, a partial metric space is a generalization of a metric space; indeed, if an axiom *p*(*x*, *x*) = 0 is imposed, then the above axioms reduce to their metric counterparts. Thus, a metric space can be defined to be a partial metric space in which each self-distance is zero.

It is clear that *p*(*x*, *y*) = 0 implies *x* = *y* from (P1) and (P2). But, *x* = *y* does not imply *p*(*x*, *y*) = 0, in general. A basic example of a partial metric space is the pair (*R* ^+^, *p*), where *p*(*x*, *y*) = max⁡{*x*, *y*} for all *x*, *y* ∈ *R* ^+^.

**Remark 3 (see [16]).**

Clearly, a limit of a sequence in a partial metric space need not be unique. Moreover, the function *p*(·, ·) need not be continuous in the sense that *x*_*n*_ → *x* and *y*_*n*_ → *y* imply *p*(*x*_*n*_, *y*_*n*_) → *p*(*x*, *y*). For example, if *X* = [0, +*∞*) and *p*(*x*, *y*) = max⁡{*x*, *y*} for *x*, *y* ∈ *X*, then for {*x*_*n*_} = {1}, *p*(*x*_*n*_, *x*) = *x* = *p*(*x*, *x*) for each *x* ≥ 1 and so, for example, *x*_*n*_ → 2 and *x*_*n*_ → 3 when *n* → *∞*.

**Proposition 4 (see [17]).**

Let *x*, *y* ∈ *X* and define the partial distance function *p* by

(6) p:X×X⟶R+kkllx,y⟼px,y=max⁡x,y,p:X×X⟶R+kkll(x,y)⟼p(x,y)=−min⁡{x,y},

For *X* = *R* ^+^ and *X* = *R* ^−^, respectively. Then, (*R* ^+^, *p*) is complete partial metric space where the self-distance for any point *x* ∈ *R* ^+^ is its value itself. The pair (*R* ^−^, *p*) is complete partial metric space for which *p* is called the usual partial metric on *R* ^−^, and where the self-distance for any point *x* ∈ *R* ^−^ is its absolute value.

**Proposition 5 (see [18]).**

If *p* is a partial metric on *X*, then the function *p* ^*s*^ defined by

(7) ps:X×X⟶R+kkkllx,y⟼psx,y=2px,y−px,x−py,y,

is a usual metric on *X*. For example, in (*R* ^−^, *p*), where *p* is the usual partial metric on *R* ^−^, we obtain the usual distance in *R* ^−^ since for any *x*, *y* ∈ *R* ^−^, *p* ^*s*^(*x*, *y*) = 2*p*(*x*, *y*) − *p*(*x*, *x*) − *p*(*y*, *y*) = *x* + *y* − 2min⁡{*x*, *y*} = |*x* − *y*|.

**Definition 6 (see [15]).**

A partial order on *X* is a binary relation ⊑ on *X* such that

(i) *x*⊑*x* (reflexivity),
(ii) if *x*⊑*y* and *y*⊑*x* then *x* = *y* (antisymmetry),
(iii) if *x*⊑*y* and *y*⊑*z* then *x*⊑*z* (transitivity).

A partially ordered set (or poset) is a pair (*X*, ⊑) such that ⊑ is a partial order on *X*. For each partial metric space (*X*, *p*) let ⊑_*p*_ be the binary relation over *X* such that *x*⊑_*p*_ *y* (to be read, *x* is part of *y*) if and only if *p*(*x*, *x*) = *p*(*x*, *y*). Then it can be shown that (*X*, ⊑_*p*_) is a poset.

For the partial metric max⁡{*a*, *b*} over the nonnegative reals, ⊑_max⁡_ is the usual ≥ ordering. For intervals, [*a*, *b*]⊑_*p*_[*c*, *d*] if and only if [*c*, *d*] is a subset of [*a*, *b*].

**Definition 7 (cf. [17–20]).**

Let (*X*, *p*) be a partial metric space and (*x*_*n*_) a sequence in (*X*, *p*). Then, we say the following:

- A sequence (*x*_*n*_) converges to a point *x* ∈ *X* if and only if *p*(*x*, *x*) = lim⁡_*n*→*∞*_ *p*(*x*_*n*_, *x*).
- A sequence (*x*_*n*_) is a* Cauchy sequence* if there exists (and is finite) lim⁡_*m*,*n*→*∞*_ *p*(*x*_*n*_, *x*_*m*_).
- A partial metric space (*X*, *p*) is said to be* complete* if every Cauchy sequence (*x*_*n*_) in *X* converges, with respect to the topology *τ*_*p*_, to a point *x* ∈ *X* such that *p*(*x*, *x*) = lim⁡_*m*,*n*→*∞*_ *p*(*x*_*m*_, *x*_*n*_). It is easy to see that every closed subset of a complete partial metric space is complete.
- A mapping *f* : *X* → *X* is called to be* continuous* at *x*_0_ ∈ *X* if for every *ɛ* > 0, there exists *δ* > 0 such that *f*(*B*_*p*_(*x*_0_, *δ*)) ⊂ *B*_*p*_(*f*(*x*_0_), *ɛ*).
- A sequence (*x*_*n*_) in a partial metric space (*X*, *p*) converges to a point *x* ∈ *X*, for any *ϵ* > 0 such that *x* ∈ *B*_*p*_(*x*, *ϵ*), there exists *n*_0_ ≥ 1 so that for any *n* ≥ *n*_0_, *x*_*n*_ ∈ *B*_*p*_(*x*, *ϵ*).

**Lemma 8 (see [18]).**

Let (*X*, *p*) be a partial metric space. Then,

(i) (*x*_*n*_) is a Cauchy sequence in (*X*, *p*) if and only if it is a Cauchy sequence in the metric space (*X*, *p* ^*s*^),
(ii) a partial metric space (*X*, *p*) is complete if and only if the metric space (*X*, *p* ^*s*^) is complete. Furthermore, lim⁡_*n*→*∞*_ *p* ^*s*^(*x*_*n*_, *x*) = 0 if and only if *p*(*x*, *x*) = lim⁡_*n*→*∞*_ *p*(*x*_*n*_, *x*) = lim⁡_*m*,*n*→*∞*_ *p*(*x*_*n*_, *x*_*m*_).

In the partial metric space (*R* ^−^, *p*), the limit of the sequence (−1/*n*) is 0 since one has lim⁡_*n*→*∞*_ *p* ^*s*^(−1/*n*, 0), where *p* ^*s*^ is the usual metric induced by *p* on *R* ^−^.

### 2.1. The Level Sets of Fuzzy Numbers

A* fuzzy number* is a fuzzy set on the real axis, that is, a mapping *u* : *R* → [0,1] which satisfies the following four conditions.

(i) *u* is normal; that is, there exists an *x*_0_ ∈ *R* such that *u*(*x*_0_) = 1.
(ii) *u* is fuzzy convex; that is, *u*[*λx* + (1 − *λ*)*y*] ≥ min⁡{*u*(*x*), *u*(*y*)} for all *x*, *y* ∈ *R* and for all *λ* ∈ [0,1].
(iii) *u* is upper semicontinuous.
(iv) The set ${\left[u\right]}_{0}=\overset{-}{\left\{x\in R:u(x)>0\right\}}$ is compact (cf. Zadeh [21]), where $\overset{-}{\left\{x\in R:u(x)>0\right\}}$ denotes the closure of the set {*x* ∈ *R* : *u*(*x*) > 0} in the usual topology of *R*.

We denote the set of all fuzzy numbers on *R* by *E* ^1^ and call it as* the space of fuzzy numbers*. *λ-level set *[*u*]_*λ*_ of *u* ∈ *E* ^1^ is defined by

(8) $$\begin{array}{c} {\left[u\right]}_{\lambda }=\left\{\begin{array}{cc} \left\{x\in R:u\left(x\right)\geq \lambda \right\}, & 0<\lambda \leq 1,\\ \overset{-}{\left\{x\in R:u(x)>\lambda \right\}}, & \lambda =0. \end{array}\right. \end{array}$$

The set [*u*]_*λ*_ is closed, bounded, and nonempty interval for each *λ* ∈ [0,1] which is defined by [*u*]_*λ*_ = [*u* ^−^(*λ*), *u* ^+^(*λ*)]. *R* can be embedded in *E* ^1^, since each *r* ∈ *R* can be regarded as a fuzzy number $\bar{r}$ defined by

(9) $$\begin{array}{c} \overset{-}{r}\left(x\right)=\left\{\begin{array}{cc} 1, & x=r,\\ 0, & x\neq r. \end{array}\right. \end{array}$$

Representation Theorem 1 (see [22]).* Let *[*u*]_*λ*_ = [*u* ^−^(*λ*), *u* ^+^(*λ*)]* for u* ∈ *E* ^1^ *and for each λ* ∈ [0,1]*. Then the following statements hold.*

(i) *u* ^−^ *is a bounded and nondecreasing left continuous function on *]0,1].
(ii) *u* ^+^ *is a bounded and nonincreasing left continuous function on *]0,1].
(iii) *The functions u* ^−^ *and u* ^+^ *are right continuous at the point λ* = 0.
(iv) *u* ^−^(1) ≤ *u* ^+^(1).

*Conversely, if the pair of functions u* ^−^ *and u* ^+^ *satisfies conditions (i)–(iv), then there exists a unique u* ∈ *E* ^1^ *such that *[*u*]_*λ*_ = [*u* ^−^(*λ*), *u* ^+^(*λ*)]* for each λ* ∈ [0,1]*. The fuzzy number u corresponding to the pair of functions u* ^−^ *and u* ^+^ *is defined by u* : *R* → [0,1], *u*(*x*) = sup⁡{*λ* : *u* ^−^(*λ*) ≤ *x* ≤ *u* ^+^(*λ*)}.

Now we give the definitions of triangular fuzzy numbers with the *λ*-level set.

**Definition 9 (triangular fuzzy number, [23, Definition, page 137]).**

The membership function *μ*_(*u*)_ of a triangular fuzzy number *u* represented by (*u*_1_, *u*_2_, *u*_3_) is interpreted, as follows:

(10) $$\begin{array}{c} \mu_{\left(u\right)}\left(x\right)=\left\{\begin{array}{cc} \frac{x-u_{1}}{u_{2}-u_{1}}, & u_{1}\leq x\leq u_{2},\\ \frac{u_{3}-x}{u_{3}-u_{2}}, & u_{2}\leq x\leq u_{3},\\ 0, & x<u_{1},\;\;x>u_{3}. \end{array}\right. \end{array}$$

Then, the result [*u*]_*λ*_ : = [*u* ^−^(*λ*), *u* ^+^(*λ*)] = [(*u*_2_ − *u*_1_)*λ* + *u*_1_, (*u*_2_ − *u*_3_)*λ* + *u*_3_] holds for each *λ* ∈ [0,1].

Let *u*, *v*, *w* ∈ *E* ^1^ and *α* ∈ *R*. Then the operations addition, scalar multiplication, and product defined on *E* ^1^ by *u* + *v* = *w*⇔[*w*]_*λ*_ = [*u*]_*λ*_ + [*v*]_*λ*_ for all  *λ* ∈ [0,1] then *w* ^−^(*λ*) = *u* ^−^(*λ*) + *v* ^−^(*λ*)  and  *w* ^+^(*λ*) = *u* ^+^(*λ*) + *v* ^+^(*λ*)  for all  *λ* ∈ [0,1].

Let *W* be the set of all closed bounded intervals *A* of real numbers with endpoints $\underline{A}$ and $\bar{A}$; that is, $A:=[\underline{A},\bar{A}]$. Define the relation *d* on *W* by $d(A,B):=\max\{|\underline{A}-\underline{B}|,|\bar{A}-\bar{B}|\}$. Then it can easily be observed that *d* is a metric on *W* (cf. Diamond and Kloeden [24]) and (*W*, *d*) is a complete metric space, (cf. Nanda [25]). Now, we can define the metric *D* on *E* ^1^ by means of the Hausdorff metric *d* as

(11) Du,v:=sup⁡λ∈[0,1]d(uλ,vλ):=sup⁡λ∈[0,1]max⁡{u−(λ)−v−(λ),u+(λ)−v+(λ)}.

**Proposition 10 (see [26]).**

Let *u*, *v*, *w*, *z* ∈ *E* ^1^ and *α* ∈ *R*. Then, the following statements hold.

(i) (*E* ^1^, *D*) is a complete metric space* (cf. Puri and Ralescu [27])*.
(ii) *D*(*αu*, *αv*) = |*α* | *D*(*u*, *v*).
(iii) *D*(*u* + *v*, *w* + *v*) = *D*(*u*, *w*).
(iv) *D*(*u* + *v*, *w* + *z*) ≤ *D*(*u*, *w*) + *D*(*v*, *z*).
(v) $\left|D(u,\bar{0})-D(v,\bar{0})|\leq D(u,v)\leq D(u,\bar{0})+D(v,\bar{0}\right)$.

**Definition 11 (see [28]).**

The following statements hold.

- A sequence *u* = (*u*_*k*_) of fuzzy numbers is a function *u* from the set *N* into the set *E* ^1^. The fuzzy number *u*_*k*_ denotes the value of the function at *k* ∈ *N* and is called as the general term of the sequence.
- A sequence (*u*_*n*_) ∈ *ω*(*F*) is called convergent to *u* ∈ *E* ^1^, if and only if for every *ɛ* > 0 there exists an *n*_0_ = *n*_0_(*ɛ*) ∈ *N* such that *D*(*u*_*n*_, *u*) < *ɛ* for all *n* ≥ *n*_0_.
- A sequence (*u*_*n*_) ∈ *ω*(*F*) is called bounded if and only if the set of its terms is a bounded set. That is to say that a sequence (*u*_*n*_) ∈ *ω*(*F*) is said to be bounded if and only if there exist two fuzzy numbers *m* and *M* such that *m*⪯*u*_*n*_⪯*M* for all *n* ∈ *N*. This means that *m* ^−^(*λ*) ≤ *u*_*n*_^−^(*λ*) ≤ *M* ^−^(*λ*) and *m* ^+^(*λ*) ≤ *u*_*n*_^+^(*λ*) ≤ *M* ^+^(*λ*) for all *λ* ∈ [0,1].

The boundedness of the sequence (*u*_*n*_) ∈ *ω*(*F*) is equivalent to the fact that

(12) $$\begin{array}{c} \underset{n\in N}{\sup}D\left(u_{n},\bar{0}\right)=\underset{n\in N}{\sup}\underset{\;\lambda \in [0,1]}{\sup}\max\left\{\left|u_{n}^{-}\left(\lambda \right)\right|,\left|u_{n}^{+}\left(\lambda \right)\right|\right\}<\infty . \end{array}$$

If the sequence (*u*_*k*_) ∈ *ω*(*F*) is bounded then the sequences of functions {*u*_*k*_^−^(*λ*)} and {*u*_*k*_^+^(*λ*)} are uniformly bounded in [0,1].

## 3. Completeness of the Sets of Sequences with Respect to the Partial Metric

Following Kadak and Ozluk [11], we give the classical sets *l*_*∞*_(*H*), *c*(*H*), *c*_0_(*H*), and *l*_*p*_(*H*) consisting of the bounded, convergent, null, and *p*-summable sequences of fuzzy level sets with the partial metric *H* ^*s*^, as follows:

(13) l∞(H)≔u=uk∈ωF:sup⁡k∈NHsuk,0¯<∞,kkkkkkkkkkk0¯∈E1u=uk∈ωF:sup⁡k∈NHsuk,0¯<∞,kc(H)≔u=uk∈ωF:lim⁡k→∞Hsuk,u=0kkkkkkkl for some u∈E1u=uk∈ωF:lim⁡k→∞Hsuk,u=0,lc0(H):=u=uk∈ωF:lim⁡k→∞Hsuk,0¯=0,llp(H):=u=(uk)∈ω(F):∑k=0∞Hsuk,0¯p<∞,kkkkkkkkkkkkkkkkkkkkkkkkkkkkkkl(1≤p<∞).

One can show that *l*_*∞*_(*H*), *c*(*H*), and *c*_0_(*H*) are complete metric spaces with the partial metric *H*_*∞*_ defined by

(14) $$\begin{array}{c} H_{\infty }\left(u,v\right):=\underset{k\in N}{\sup}\left\{H^{s}\left(u_{k},v_{k}\right)\right\}, \end{array}$$

where *u* = (*u*_*k*_) and *v* = (*v*_*k*_) are the elements of the sets *c*(*H*), *c*_0_(*H*), or *l*_*∞*_(*H*). Also, the space *l*_*p*_(*H*) is complete metric space with the partial metric *H*_*p*_ defined by

(15) $$\begin{array}{c} H_{p}\left(u,v\right):={\left\{\overset{\infty }{\underset{k=0}{\sum }}H^{s}{\left(u_{k},v_{k}\right)}^{p}\right\}}^{1/p},\;\left(1\leq p<\infty \right), \end{array}$$

where *u* = (*u*_*k*_) and *v* = (*v*_*k*_) are the points of *l*_*p*_(*H*).

**Theorem 12 .**

Let *μ*(*H*) denote any of the spaces *bs*(*H*), *cs*(*H*), and *cs*_0_(*H*), and *u* = (*u*_*k*_), *v* = (*v*_*k*_) ∈ *μ*(*H*). Define the partial distance function *H*_*∞*_^*p*^ on *μ*(*H*) by

(16) H∞p:μ(H)×μ(H)⟶R+kkkkkkkkkkkllu,v⟶H∞pu,v:=sup⁡n∈N∑k=0nHsuk,vk.

Then, (*μ*(*H*), *H*_*∞*_^*p*^) is a complete metric space.

**Proof**

Since the proof is similar for the spaces *cs*(*H*) and *cs*_0_(*H*), we prove the theorem only for the space *bs*(*H*). Let *u* = (*u*_*k*_), *v* = (*v*_*k*_), and *w* = (*w*_*k*_) ∈ *bs*(*H*). Then,

(i) by using the axiom (P1) in Definition 1, it is trivial that
  (17) u=v⟺H∞p(u,v)=sup⁡n∈N{∑k=0nHsuk,vk}kkkkkkkkkkkkklkl=sup⁡n∈N∑k=0n2H(uk,vk)−H(uk,uk)kkkkkkkkkkkkkkkkkkkkkkk−H(vk,vk)=0kkl⟺sup⁡n∑k=0n{Hs(uk,uk)}=sup⁡n∑k=0n{Hs(vk,vk)}=0kkl⟺H∞p(u,v)=H∞p(u,u)=H∞p(v,v).
(ii) By using the axiom (P2) in Definition 1, it follows that
  (18) H∞pu,u=sup⁡n∈N∑k=0nHsuk,uk≤sup⁡n∈N∑k=0nHsuk,vk=H∞pu,v.
(iii) By using the axiom (P3) in Definition 1, it is clear that
  (19) H∞pu,v=sup⁡n{∑k=0nHs(uk,vk)}=sup⁡n{∑k=0nHs(vk,uk)}=H∞p(v,u).
(iv) By using the axiom (P4) in Definition 1 with the inequalities *H*(*u*_*k*_, *w*_*k*_) ≤ *H*(*u*_*k*_, *v*_*k*_) + *H*(*v*_*k*_, *w*_*k*_) − *H*(*v*_*k*_, *v*_*k*_) and *H* ^*s*^(*u*_*k*_, *u*_*k*_) = 0, we have
  (20) H∞pu,w =sup⁡n∑k=0nHs(uk,wk):k∈N =sup⁡n∑k=0n2H(uk,wk)−H(uk,uk)−H(wk,wk) ≤sup⁡n∑k=0n2[H(uk,vk)+H(vk,wk)−H(vk,vk)]kkkkkkkkkkk−Huk,uk−Hwk,wk =sup⁡n∑k=0n2Huk,vk−Huk,uk−Hvk,vkkkkkkkkkkkk+2Hvk,wk−Hvk,vk−Hwk,wk ≤sup⁡n∑k=0n2H(uk,vk)−H(uk,uk)−H(vk,vk)  +sup⁡n∑k=0n2H(vk,wk)−H(vk,vk)−H(wk,wk) =sup⁡n∑k=0nHsuk,vk+sup⁡n∑k=0nHsvk,wk  −sup⁡n∑k=0nHsvk,vk =H∞p(u,v)+H∞p(v,w)−H∞p(v,v).

Therefore, one can conclude that (*bs*(*H*), *H*_*∞*_^*p*^) is a partial metric space on *bs*(*H*). It remains to prove the completeness of the space *bs*(*H*).  Let (*u*_*m*_) be any Cauchy sequence on *bs*(*H*), where *u*_*m*_ = {*u*_1_^(*m*)^, *u*_2_^(*m*)^,…}. Then, for any *ϵ* > 0, there exists *N* ∈ *N* for all *m*, *r* > *N* such that

(21) $$\begin{array}{c} H_{\infty }^{p}\left(u_{m},u_{r}\right)=\underset{n\in N}{\sup}\left\{\overset{n}{\underset{k=0}{\sum }}H^{s}\left(u_{k}^{\left(m\right)},u_{k}^{\left(r\right)}\right)\right\}<\epsilon . \end{array}$$

A fortiori, for every fixed *k* ∈ *N* and for *m*, *r* > *N*

(22) $$\begin{array}{c} H^{s}\left(\overset{n}{\underset{k=0}{\sum }}u_{k}^{\left(m\right)},\overset{n}{\underset{k=0}{\sum }}u_{k}^{\left(r\right)}\right)<\epsilon . \end{array}$$

Hence for every fixed *k* ∈ *N*, by using the completeness of (*E* ^1^, *H* ^*s*^) in Theorem 3.1 [11], we say the sequence (*u*_*k*_^(*m*)^) = {*u*_*k*_^(1)^, *u*_*k*_^(2)^,…} is a Cauchy sequence and is uniformly convergent. Now, we suppose that lim⁡_*m*→*∞*_ *u*_*k*_^(*m*)^ = *u*_*k*_ and *u* = (*u*_1_, *u*_2_,…). We must show that

(23) $$\begin{array}{c} \underset{m\to \infty }{\lim}H_{\infty }^{p}\left(u_{m},u\right)=0,\;u\in bs\left(H\right). \end{array}$$

The constant *N* ∈ *N* for all *m* > *N*, taking the limit for *r* → *∞* in (22), we obtain

(24) $$\begin{array}{c} H^{s}\left(\overset{n}{\underset{k=0}{\sum }}u_{k}^{\left(m\right)},\overset{n}{\underset{k=0}{\sum }}u_{k}\right)<\epsilon , \end{array}$$

for all *k* ∈ *N*. Since (*u*_*k*_^(*m*)^) ∈ *bs*(*H*), there exists a number $M≻\bar{0}$ such that $H^{s}(\sum_{k=0}^{n}u_{k}^{\left(m\right)},\bar{0})\leq M$ for all *k* ∈ *N*. Thus, (24) gives together with the triangle inequality of partial metric for *m* > *N* that

(25) Hs∑k=0nuk,0¯ ≤Hs∑k=0nuk,∑k=0nuk(m)+Hs∑k=0nuk(m),∑k=0n0¯  −Hs∑k=0nuk(m),∑k=0nuk(m)≤ϵ+M.

It is clear that (25) holds for every *k* ∈ *N* whose right-hand side does not involve *k*. This leads us to the consequence that *u* = (*u*_1_, *u*_2_,…) is bounded sequence of fuzzy numbers hence *u* ∈ *bs*(*H*). Also, from (24) we obtain for *m* > *N* that

(26) $$\begin{array}{c} H_{\infty }^{p}\left(u_{m},u\right)=\underset{k\in N}{\sup}\left\{\overset{n}{\underset{k=0}{\sum }}H^{s}\left(u_{k}^{\left(m\right)},u_{k}\right)\right\}\leq \epsilon . \end{array}$$

This shows that (23) holds and lim⁡_*m*_ *H*_*∞*_^*p*^(*u*_*m*_, *u*) = 0. Since (*u*_*m*_) is an arbitrary Cauchy sequence, *bs*(*H*) is complete.

**Theorem 13 .**

Define the distance functions *H*_Δ_(*u*, *v*), *H*_*q*_^Δ^(*u*, *v*), and *H*_*∞*_^Δ^(*u*, *v*) by

(27) HΔ(u,v):=∑k=0∞{Hs[Δuk,Δvk]},HqΔ(u,v):=∑k=0∞HsΔuk,Δvkq1/q,H∞Δ(u,v):=sup⁡k∈N{Hs[Δuk,Δvk]},

where *u* = (*u*_*k*_), *v* = (*v*_*k*_) are the element of the spaces *bv*(*H*), *bv*_*q*_(*H*), or *bv*_*∞*_(*H*), respectively. Then, (*bv*(*H*), *H* ^Δ^), (*bv*_*q*_(*H*), *H*_*q*_^Δ^), and (*bv*_*∞*_(*H*), *H*_*∞*_^Δ^) are complete metric spaces.

**Proof**

Since the proof is similar for the spaces *bv*(*H*) and *bv*_*∞*_(*H*), we prove the theorem only for the space *bv*_*q*_(*H*). One can easily establish that *H*_*q*_^Δ^ defines a metric on *bv*_*q*_(*H*). Let *x* ^*i*^ = {*x*_0_^(*i*)^, *x*_1_^(*i*)^,…} be any Cauchy sequence on *bv*_*q*_(*H*). Then for every *ϵ* > 0, there exists a positive integer *n*_0_(*ϵ*) ∈ *N* for all *i*, *j* > *n*_0_, such that

(28) $$\begin{array}{c} H_{q}^{\Delta }\left(x^{i},x^{j}\right):=\overset{\infty }{\underset{n=0}{\sum }}{\left\{H^{s}{\left[{\left(\Delta x\right)}_{n}^{i},{\left(\Delta x\right)}_{n}^{j}\right]}^{q}\right\}}^{1/q}<\epsilon , \end{array}$$

where (Δ*x*)_*n*_ = *x*_*n*_ − *x*_*n*−1_ and $x_{-1}=\bar{0}$. We obtain for each fixed *n* ∈ *N* from (28) that

(29) $$\begin{array}{c} H^{s}\left[{\left(\Delta x\right)}_{n}^{i},{\left(\Delta x\right)}_{n}^{j}\right]<\epsilon , \end{array}$$

for all *i*, *j* > *n*_0_(*ϵ*), which leads us to the fact that the sequence {(Δ*x*)_*n*_^*i*^} is a Cauchy sequence and is convergent. Now, we suppose that (Δ*x*)_*n*_^*i*^ → (Δ*x*)_*n*_ as *n* → *∞*. We have from (29) for each *m* ∈ *N* and *i*, *j* > *n*_0_(*ϵ*), that

(30) $$\begin{array}{c} \overset{m}{\underset{k=0}{\sum }}H^{s}{\left[{\left(\Delta x\right)}_{k}^{i},{\left(\Delta x\right)}_{k}^{j}\right]}^{q}\leq H_{q}^{\Delta }{\left(x^{i},x^{j}\right)}^{q}<\epsilon^{q}. \end{array}$$

Take any *i* > *n*_0_(*ϵ*). Let firstly *j* → *∞* and nextly *m* → *∞* in (30) to obtain *H*_*q*_^Δ^(*x* ^*i*^, *x*) ≤ *ϵ*. Finally, by using Minkowski's inequality for each *m* ∈ *N*

(31) ∑k=0mHsΔxk,0¯q1/q≤HqΔ(xi,x)+HqΔ(xi,0¯)≤ϵ+HqΔ(xi,0¯)<∞,

which implies that *x* ∈ *bv*_*q*_(*H*). Since *H*_*q*_^Δ^(*x* ^*i*^, *x*) ≤ *ϵ* for all *i* > *n*_0_(*ϵ*), it follows that *x* ^*i*^ → *x* as *i* → *∞*. Since (*x* ^*i*^) is an arbitrary Cauchy sequence, the space *bv*_*q*_(*H*) is complete. This step concludes the proof.

## 4. The Duals of the Sets of Sequence with the Partial Metric

The idea of dual sequence space, which plays an important role in the representation of linear functionals and the characterization of matrix transformations between sequence spaces, was introduced by Köthe and Toeplitz [29], whose main results concerned *α*-duals. An account of the duals of sequence spaces can be found in Köthe [30]. One can also find about different types of duals of sequence spaces in Maddox [31].

In this section, we focus on the alpha-, beta- and gamma-duals of the classical sets of sequences of fuzzy numbers with partial metric. For the sets *λ*(*H*), *μ*(*H*), and *S*(*λ*(*H*), *μ*(*H*)) of sequences defined by

(32) SλH,μH≔w=wk∈ωF:wkzk∈μHkkkkk∀z=(zk)∈λ(H),

is called the multiplier sets of *λ*(*H*) and *μ*(*H*) for all *k* ∈ *N*. One can easily observe for a sequence set *ν*(*H*) of fuzzy level sets that the inclusions

(33) SλH,μH⊂SνH,μH if   νH⊂λH,SλH,μH⊂SλH,νH if   μH⊂νH,

hold. The alpha-, beta- and gamma-duals {*λ*(*H*)}^*α*^, {*λ*(*H*)}^*β*^, and {*λ*(*H*)}^*γ*^ of a set *λ*(*H*) ⊂ *ω*(*F*) are, respectively, defined by

(34) λ(H)α:=w=(wk)∈ω(F):(wkzk)∈l1(H)kkkkkkkkkkkk∀z=(zk)∈λ(H),λ(H)β:=w=(wk)∈ω(F):(wkzk)∈cs(H)kkkkkkkkkkkk∀z=zk∈λH,λ(H)γ:=w=(wk)∈ω(F):(wkzk)∈bs(H)kkkkkkkkkkkk∀z=(zk)∈λ(H),

where (*w*_*k*_ *z*_*k*_) the coordinatewise product of the sequences *w* and *z* of level sets for all *k* ∈ *N*. Then {*λ*(*H*)}^*β*^ is called *β*-dual of *λ*(*H*) or the set of all factor sequences of *λ*(*H*) are in *cs*(*H*). Firstly, we give a remark concerning with the convergence factor sequences of fuzzy level sets with partial metric.

**Remark 14 .**

Let *∅* ≠ *λ*(*H*) ⊂ *ω*(*F*). Then the following statements are valid.

(a) {*λ*(*H*)}^*β*^ is a set of sequence and *φ*(*F*)<{*λ*(*H*)}^*β*^ < *ω*(*F*) (“<” stands for “is a linear subset of ”) where
  (35) $$\begin{array}{c} \varphi \left(F\right):=\left\{u=\left(u_{k}\right):\exists N\in N,\;\;\forall k\geq N,\;\;u_{k}=\bar{0}\right\}. \end{array}$$
(b) If *λ*(*H*) ⊂ *μ*(*H*) ⊂ *ω*(*F*) then {*μ*(*H*)}^*β*^ < {*λ*(*H*)}^*β*^.
(c) *λ*(*H*)⊂{*λ*(*H*)}^*ββ*^ : = ({*λ*(*H*)}^*β*^)^*β*^.
(d) {*φ*(*F*)}^*β*^ = *ω*(*F*) and {*ω*(*F*)}^*β*^ = *φ*(*F*).

**Proof**

Since the proof is trivial for conditions (b) and (c), we prove only (a) and (d). Let *m* = (*m*_*k*_) and *n* = (*n*_*k*_)∈{*λ*(*H*)}^*β*^.

(a) Let *l* ∈ *λ*(*H*). Then we get (*m*_*k*_ *l*_*k*_) ∈ *cs*(*H*); (*n*_*k*_ *l*_*k*_) ∈ *cs*(*H*) and (*m*_*k*_ + *n*_*k*_)*l*_*k*_ = (*m*_*k*_ *l*_*k*_)+(*n*_*k*_ *l*_*k*_) ∈ *cs*(*H*). Since *l* is arbitrary, *m* + *n* ∈ {*λ*(*H*)}^*β*^. For any *α* ∈ *R* and *w* = (*w*_*k*_)∈{*λ*(*H*)}^*β*^ we have
  (36) $$\begin{array}{c} \left(\alpha w_{k}\right)l_{k}=\alpha \left(w_{k}l_{k}\right)\in cs\left(H\right), \end{array}$$
  and we get *αw* ∈ {*λ*(*H*)}^*β*^. Therefore, {*λ*(*H*)}^*β*^ is a linear subset of *ω*(*F*).
(d) Using (a) we need only show {*ω*(*F*)}^*β*^ ⊂ *φ*(*F*). Suppose that *w* = (*w*_*n*_)∈{*ω*(*F*)}^*β*^ and *z* = (*z*_*n*_) be given with geometric division by *z*_*n*_ : = (1/*w*_*n*_) if $w_{n}\neq \bar{0}$ and $z_{n}:=\bar{0}$ otherwise. By taking into account the set *φ*(*F*) from the case (a), then there exists an integer *N* ∈ *N* for all *n* ≥ *N* such that $w_{n}=\bar{0}$. Thus, we have
  (37) $$\begin{array}{c} \overset{\infty }{\underset{n=0}{\sum }}w_{n}z_{n}=\underset{n}{\sum }1\;\left(w_{n}\neq \bar{0}\right). \end{array}$$
  Further, (*w*_*n*_ *z*_*n*_) ∈ *cs*(*H*) implies that *w* ∈ *φ*(*F*). The rest is an immediate consequence of this part; we omit the detail.

**Theorem 15 .**

The following statements hold.

- {*c*_0_(*H*)}^*β*^ = {*c*(*H*)}^*β*^ = {*l*_*∞*_(*H*)}^*β*^ = *l*_1_(*H*).
- {*l*_1_(*H*)}^*β*^ = *l*_*∞*_(*H*).

**Proof**

(a) Obviously {*l*_*∞*_(*H*)}^*β*^ ⊂ {*c*(*H*)}^*β*^ ⊂ {*c*_0_(*H*)}^*β*^ by Remark 14(b). Then we must show that *l*_1_(*H*)⊂{*l*_*∞*_(*H*)}^*β*^ and {*c*_0_(*H*)}^*β*^ ⊂ *l*_1_(*H*). Now, consider *w* = (*w*_*k*_) ∈ *l*_1_(*H*) and *z* = (*z*_*k*_) ∈ *l*_*∞*_(*H*) are given. Then

(38) $$\begin{array}{c} \overset{n}{\underset{k=0}{\sum }}H^{s}\left(w_{k}z_{k},\bar{0}\right)\leq \underset{k}{\sup}\;H^{s}\left(z_{k},\bar{0}\right)\overset{n}{\underset{k=0}{\sum }}H^{s}\left(w_{k},\bar{0}\right)<\infty , \end{array}$$

which implies that *wz* ∈ *cs*(*H*). So the condition *l*_1_(*H*)⊂{*l*_*∞*_(*H*)}^*β*^ holds.

Conversely, for a given *y* = (*y*_*k*_) ∈ *ω*(*F*)∖*l*_1_(*H*) we prove the existence of an *x* ∈ *c*_0_(*H*) with *yx* ∉ *cs*(*H*). According to *y* ∉ *l*_1_(*H*) we may take an index sequence (*n*_*p*_) which is a strictly increasing real valued sequence with *n*_0_ = 0 and $\sum_{k=n_{p-1}}^{n_{p}-1}H^{s}(y_{k},\bar{0})>p\;\;(p\in N)$. If we define *x* = (*x*_*k*_) ∈ *c*_0_(*H*) by *x*_*k*_ : = ((sgn⁡*y*_*k*_)/*p*), where the real signum function defined by

(39) $$\begin{array}{c} \mathrm{sgn}\left(u\right):=\left\{\begin{array}{cc} \frac{u}{\left|u\right|}, & u\neq \bar{0},\\ 0, & u=\bar{0}, \end{array}\right. \end{array}$$

for all *u* = (*u*_*k*_) ∈ *E* ^1^, thus, we get

(40) $$\begin{array}{c} \overset{n_{p}-1}{\underset{k=n_{p-1}}{\sum }}\left(y_{k}x_{k}\right)=\frac{1}{p}\overset{n_{p}-1}{\underset{k=n_{p-1}}{\sum }}y_{k}\left(\mathrm{sgn}y_{k}\right)=\frac{1}{p}\overset{n_{p}-1}{\underset{k=n_{p-1}}{\sum }}H^{s}\left(y_{k},\bar{0}\right)\geq 1, \end{array}$$

for all *n*_*p*−1_ ≤ *k* < *n*_*p*_. Therefore *yx* ∉ *cs*(*H*) and thus *y* ∉ {*c*_0_(*H*)}^*β*^. Hence {*c*_0_(*H*)}^*β*^ ⊂ *l*_1_(*H*).

(b) From the condition (c) of Remark 14 we have *l*_*∞*_(*H*)⊂({*l*_*∞*_(*H*)}^*β*^)^*β*^ = {*l*_1_(*H*)}^*β*^ since {*l*_*∞*_(*H*)}^*β*^ = *l*_1_(*H*). Now we assume the existence of a *w* = (*w*_*n*_)∈{*l*_1_(*H*)}^*β*^∖*l*_*∞*_(*H*). Since *w* is an unbounded sequence there exists a subsequence (*w*_*n*_*k*__) of (*w*_*n*_) such that $H^{s}(w_{n_{k}},\bar{0})\geq {\left(k+1\right)}^{2}$ for all *k* ∈ *N*_1_. The sequence (*x*_*n*_) is defined by *x*_*n*_ : = (sgn⁡(*w*_*n*_*k*__)/(*k* + 1)^2^) if *n* = *n*_*k*_ and $\bar{0}$ otherwise. Then *x* ∈ *l*_1_(*H*). However

(41) $$\begin{array}{c} \underset{n}{\sum }w_{n}x_{n}=\underset{k}{\sum }\frac{H^{s}\left(w_{n_{k}},\bar{0}\right)}{{\left(k+1\right)}^{2}}\geq \underset{k}{\sum }1=\infty . \end{array}$$

Hence *w* ∉ {*l*_1_(*H*)}^*β*^, which contradicts our assumption and {*l*_1_(*H*)}^*β*^ ⊂ *l*_*∞*_(*H*). This step completes the proof.

Further to the statements in Remark 14 we make the following remarks which are immediate consequences of the definition of the *ζ*-duals (*ζ* ∈ {*α*, *β*, *γ*}).

**Remark 16 .**

Let *∅* ≠ *λ*(*H*) ⊂ *ω*(*F*). Then the following statements are valid.

(a) *φ*(*F*)<{*λ*(*H*)}^*α*^ < {*λ*(*H*)}^*β*^ < {*λ*(*H*)}^*γ*^ < *ω*(*F*); in particular, {*λ*(*H*)}^*ζ*^ is a set of sequence.
(b) If *λ*(*H*) < *μ*(*H*) < *ω*(*F*) then {*μ*(*H*)}^*ζ*^ < {*λ*(*H*)}^*ζ*^.
(c) If *I* is an index set, if *λ*(*H*)_*i*_ are sets of sequences and if *λ*(*H*): = ⋃_*i*∈*I*_ *λ*(*H*)_*i*_, then
  (42) $$\begin{array}{c} {\langle \lambda (H)\rangle }^{\zeta }=\underset{i\in I}{⋂}{\left\{\lambda {\left(H\right)}_{i}\right\}}^{\zeta }, \end{array}$$
  where the notation “〈〉” stands for the span of linear subset in *R*.
(d) If *λ*(*H*)<{*λ*(*H*)}^*ζζ*^ : = ({*λ*(*H*)}^*ζ*^)^*ζ*^. (*ζ* ∈ {*α*, *β*, *γ*})

**Proof**

Condition (b) is obviously true, and (a) follows from *l*_*∞*_(*H*) < *cs*(*H*) < *bs*(*H*). We only show conditions (c) and (d) taking *ζ* = *α*. Other parts can be obtained in a similar way.

(c) Now, as an immediate consequence *λ*(*H*)_*i*_ ⊂ 〈*λ*(*H*)〉 that the following conditions
  (43) $$\begin{array}{c} {\langle \lambda (H)\rangle }^{\alpha }\subset {\left\{\lambda {\left(H\right)}_{i}\right\}}^{\alpha },\;{\langle \lambda (H)\rangle }^{\alpha }\subset \underset{i\in I}{⋂}{\left\{\lambda {\left(H\right)}_{i}\right\}}^{\alpha }, \end{array}$$
 hold by (b). On the other hand, if *y* ∈ ⋂_*i*∈*I*_{*λ*(*H*)_*i*_}^*α*^, that is *y* ∈ {*λ*(*H*)_*i*_}^*α*^, then *xy* ∈ *l*_1_(*H*) for all *x* ∈ *λ*(*H*) and therefore *y* ∈ {*λ*(*H*)}^*α*^ ⊂ 〈*λ*(*H*)〉^*α*^.
(d) We prove *λ*(*H*)<{*λ*(*H*)}^*αα*^. Let *w* ∈ *λ*(*H*); then, *wz* ∈ *l*_1_(*H*) for all *z* ∈ {*λ*(*H*)}^*α*^; thus, *w* ∈ {*λ*(*H*)}^*αα*^ and *λ*(*H*)<{*λ*(*H*)}^*αα*^ by (a).

In general *λ*(*H*)≠{*λ*(*H*)}^*ζζ*^ as we get from Theorem 15(a) in the case of *ζ* = *β* and *λ*(*H*): = *c*_0_(*H*). We have {*c*_0_(*H*)}^*ββ*^ = *l*_*∞*_(*H*) ≠ *c*_0_(*H*). This remark gives rise to the following definition.

**Definition 17 (*ζ*-space, Köthe space).**

Let *ζ* ∈ {*α*, *β*, *γ*}, and let *λ*(*H*) be a set of sequence. *λ*(*H*) is called *ζ*-space if *λ*(*H*) = {*λ*(*H*)}^*ζζ*^. Further, an *α*-space is also called a Köthe space or perfect sequence space.

From Remarks 4.3(d) and (b) we obtain immediately the following remark.

**Remark 18 .**

If *λ*(*H*) is a set of sequence over real field and (*ζ* ∈ {*α*, *β*, *γ*}), then {*λ*(*H*)}^*ζ*^ is a *ζ*-space; that is, {*λ*(*H*)}^*ζ*^ = {*λ*(*H*)}^*ζζζ*^.

Now we look for sufficient conditions for {*λ*(*H*)}^*α*^ = {*λ*(*H*)}^*β*^ = {*λ*(*H*)}^*γ*^. This gives rise to the notion of solidity.

**Definition 19 (Solidness).**

Let *λ*(*H*) be a set of sequence over the field *R*. Then *λ*(*H*) is called solid if

(44) u=(uk)∈ω(F):∃(xk)∈λ(H)  ∀k∈N:Hs(uk,0¯)kkk≤Hs(xk,0¯)⊂λ(H).

**Theorem 20 .**

Consider *λ*(*H*) < *ω*(*F*) is any set of sequence over the field *R*; then, the following statements hold.

- If *λ*(*H*) is a Köthe space, then *λ*(*H*) is solid.
- If *λ*(*H*) is solid, then {*λ*(*H*)}^*α*^ = {*λ*(*H*)}^*β*^ = {*λ*(*H*)}^*γ*^.
- If *λ*(*H*) is a Köthe space, then *λ*(*H*) is a *ζ*-space.

**Proof**

Let *λ*(*H*) < *ω*(*H*) be a set of sequence over the field *R*.

(a) If *λ*(*H*) is a Köthe space and *u* ∈ *ω*(*F*), then *u* ∈ {*λ*(*H*)}^*αα*^ if and only if the condition *uz* ∈ *l*_1_(*H*) holds for all *z* ∈ {*λ*(*H*)}^*α*^. Besides this we obtain $H^{s}(v_{k},\bar{0})\leq H^{s}(u_{k},\bar{0})$ for *u* = (*u*_*k*_) ∈ *λ*(*H*) and *v* = (*v*_*k*_) ∈ *ω*(*F*) and the statement
  (45) $$\begin{array}{c} \underset{k}{\sum }H^{s}\left(v_{k}z_{k},\bar{0}\right)\leq \underset{k}{\sum }H^{s}\left(u_{k}z_{k},\bar{0}\right)<\infty , \end{array}$$
  holds for each *z* ∈ {*λ*(*H*)}^*α*^. Therefore *vz* ∈ *l*_1_(*H*). Hence *v* ∈ *λ*(*H*) and *λ*(*H*) is solid over the real field.
(b) Consider *λ*(*H*) is solid. To show {*λ*(*H*)}^*α*^ = {*λ*(*H*)}^*β*^ = {*λ*(*H*)}^*γ*^, it suffices to verify {*λ*(*H*)}^*γ*^ < {*λ*(*H*)}^*α*^ as we have Remark 16 (a). So, let *v* = (*v*_*k*_)∈{*λ*(*H*)}^*γ*^; that is,
  (46) $$\begin{array}{c} \underset{n\in N}{\sup}\left\{\overset{n}{\underset{k=0}{\sum }}H^{s}\left(u_{k}v_{k},\bar{0}\right)\right\}<\infty \;\text{for}\;\text{every}\;\;u=\left(u_{k}\right)\in \lambda \left(H\right). \end{array}$$
  By taking into account solidness of *λ*(*H*), for *z* = (*z*_*k*_) ∈ *λ*(*H*), where *z*_*k*_ = *u*_*k*_sgn⁡(*u*_*k*_ *v*_*k*_) and the condition $H^{s}(z_{k},\bar{0})\leq H^{s}(u_{k},\bar{0})$ holds; there exists a sequence *u* = (*u*_*k*_) ∈ *λ*(*H*) for all *k* ∈ *N*. Therefore by combining this with the inclusion (46) we deduce that the condition
  (47) ∑k=0NHsukvk,0¯=∑k=0Nvkuksgn⁡(ukvk)=Hs∑k=0Nvkzk,0¯≤sup⁡n∈NHs∑k=0nvkzk,0¯<∞,
  holds and *uv* ∈ *l*_1_(*H*). Hence *v* ∈ {*λ*(*H*)}^*α*^ and {*λ*(*H*)}^*γ*^ < {*λ*(*H*)}^*α*^.
(c) This is an obvious consequence of Remark 18 and conditions (a), (b) in Theorem 20.

**Theorem 21 .**

The following statements hold.

- The sets *φ*(*F*), *ω*(*F*), *l*_*p*_(*H*), *c*_0_(*H*), and *l*_*∞*_(*H*) of sequences are solid.
- The sets *c*(*H*) and *bv*(*H*) of sequences are not solid; therefore, none of them is a Köthe space.
- For each *ζ* ∈ {*α*, *β*, *γ*}, then
  - {*l*_1_(*H*)}^*ζ*^ = *l*_*∞*_(*H*) and {*l*_*∞*_(*H*)}^*ζ*^ = *l*_1_(*H*),
  - {*ω*(*F*)}^*ζ*^ = *φ*(*F*) and {*φ*(*F*)}^*ζ*^ = *ω*(*F*).
- If *ζ* ∈ {*α*, *β*, *γ*} and *c*_0_(*H*) < *μ*(*H*) < *l*_*∞*_(*H*), then {*μ*(*H*)}^*ζ*^ = *l*_1_(*H*) and *μ*(*H*)⊂{*μ*(*H*)}^*ζζ*^ = *l*_*∞*_(*H*). In particular {*c*_0_(*H*)}^*ζ*^ = {*c*(*H*)}^*ζ*^ = *l*_1_(*H*), and each of *c*(*H*), *c*_0_(*H*) is not a *ζ*-space.

**Proof**

Given specified sets are solid in (a) and (b) is an immediate consequence of their definition. Additionally, the parts (i) and (ii) of (c) can be obtained Theorem 15 and Remark 14(d). Since *c*_0_(*H*) and *l*_*∞*_(*H*) are solid, we know that {*c*_0_(*H*)}^*ζ*^ = {*l*_*∞*_(*H*)}^*ζ*^ = *l*_1_(*H*). So the statements in (d) obtain from Remark 16(b).

Next, we determine the *ζ*-duals of the spaces *cs*(*H*), *bs*(*H*), *bv*(*H*), and *bv*_0_(*H*). We will find that none of these sets is solid; in particular, none of them is a Köthe space.

**Theorem 22 .**

The following statements hold.

- {*cs*(*H*)}^*α*^ = {*bv*(*H*)}^*α*^ = {*bv*_0_(*H*)}^*α*^ = {*bs*(*H*)}^*α*^ = *l*_1_(*H*).
- {*cs*(*H*)}^*β*^ = *bv*(*H*), {*bv*(*H*)}^*β*^ = *cs*(*H*), {*bv*_0_(*H*)}^*β*^ = *bs*(*H*), {*bs*(*H*)}^*β*^ = *bv*_0_(*H*).
- {*cs*(*H*)}^*γ*^ = *bv*(*H*), {*bv*(*H*)}^*γ*^ = *bs*(*H*), {*bv*_0_(*H*)}^*γ*^ = *bs*(*H*), {*bs*(*H*)}^*γ*^ = *bv*(*H*).

In particular the sets *cs*(*H*), *bs*(*H*), *bv*(*H*), and *bv*_0_(*H*) of sequences are *β*-spaces, but they are not Köthe spaces. Moreover, the sets *bs*(*H*) and *bv*(*H*) of sequences are *γ*-spaces, whereas both *cs*(*H*) and *bv*_0_(*H*) are not *γ*-spaces. None of the spaces *cs*(*H*), *bs*(*H*), *bv*(*H*), and *bv*_0_(*H*) is solid.

**Proof**

We prove the cases for the spaces {*cs*(*H*)}^*ζ*^, *ζ* ∈ {*α*, *β*, *γ*} and the proofs of all other cases are quite similar.

(a) Let *x* = (*x*_*k*_) ∈ *cs*(*H*) and *y* = (*y*_*k*_) ∈ *l*_1_(*H*). Then,
  (48) ∑kHsykxk,0¯≤sup⁡kHs(xk,0¯)∑kHs(yk,0¯)<∞kkkkkkkkkkkkkkkkkkkkkk∀k∈N.
 Therefore, *y* ∈ {*cs*(*H*)}^*α*^ which gives that *l*_1_(*H*)⊂{*cs*(*H*)}^*α*^.
 Conversely, suppose that *y* = (*y*_*k*_)∈{*cs*(*H*)}^*α*^∖*l*_1_(*H*). Then we can construct an index sequence (*n*_*p*_) with *n*_*p*_ < *n*_*p*+1_ and $\sum_{k=n_{p}+1}^{n_{p+1}}H^{s}(y_{k},\bar{0})>4^{p}$. Define *x* = (*x*_*k*_) by
  (49) $$\begin{array}{c} x_{k}:=\left\{\begin{array}{cc} {\left(-1\right)}^{k}2^{-p}, & n_{p}<k\leq n_{p+1},\\ 0, & \text{otherwise}. \end{array}\right. \end{array}$$
 Then *x* = (*x*_*k*_) ∈ *cs*(*H*). According to the choice of *n*_*p*_ the inequalities
  (50) $$\begin{array}{c} \underset{k}{\sum }H^{s}\left(y_{k}x_{k},\bar{0}\right)\geq \underset{p}{\sum }2^{-p}\overset{n_{p+1}}{\underset{k=n_{p}+1}{\sum }}H^{s}\left(y_{k},\bar{0}\right)\geq \underset{p}{\sum }2^{p}, \end{array}$$
 hold. Thus *xy* ∉ *l*_1_(*H*), which implies *y* ∉ {*cs*(*H*)}^*α*^. This contradicts that *y* ∈ {*cs*(*H*)}^*α*^. Therefore {*cs*(*H*)}^*α*^ ⊂ *l*_1_(*H*).
 As well if we take the sequence (*x*_*k*_) by
  (51) $$\begin{array}{c} x_{k}:=\left\{\begin{array}{cc} 2^{-p}, & n_{p}<k\leq n_{p+1},\\ 0, & \text{otherwise}, \end{array}\right. \end{array}$$

 the condition {*bv*_0_(*H*)}^*α*^ ⊂ *l*_1_(*H*) holds.
(b) Let *u* = (*u*_*k*_)∈{*cs*(*H*)}^*β*^ and *w* = (*w*_*k*_) ∈ *c*_0_(*H*). Define the sequence *v* = (*v*_*k*_) ∈ *cs*(*H*) by *v*_*k*_ = (*w*_*k*_ − *w*_*k*+1_) for all *k* ∈ *N*. Therefore, ∑_*k*_ *u*_*k*_ *v*_*k*_ converges, but
  (52) $$\begin{array}{c} \overset{n}{\underset{k=0}{\sum }}\left(w_{k}-w_{k+1}\right)u_{k}=\left[\overset{n-1}{\underset{k=1}{\sum }}w_{k}\left(u_{k}-u_{k-1}\right)\right]-w_{n+1}u_{n}, \end{array}$$
 and the inclusion *l*_1_(*H*) ⊂ *cs*(*H*) yields that (*u*_*k*_)∈{*cs*(*H*)}^*β*^ ⊂ {*l*_1_(*H*)}^*β*^ = *l*_*∞*_(*H*). Then we derive by passing to the limit in (52) as *n* → *∞* which implies that
  (53) $$\begin{array}{c} \overset{\infty }{\underset{k=0}{\sum }}\left(w_{k}-w_{k+1}\right)u_{k}=\overset{\infty }{\underset{k=0}{\sum }}w_{k}\left(u_{k}-u_{k-1}\right), \end{array}$$
 for every *k* ∈ *N*. Hence (*u*_*k*_ − *u*_*k*−1_)∈{*c*_0_(*H*)}^*β*^ = {*c*_0_(*H*)}^*α*^ = *l*_1_(*H*); that is, *u* ∈ *bv*(*H*). Therefore, {*cs*(*H*)}^*β*^⊆*bv*(*H*).
 Conversely, suppose that *u* = (*u*_*k*_) ∈ *bv*(*H*). Then, (*u*_*k*_ − *u*_*k*−1_) ∈ *l*_1_(*H*). Further, if *v* = (*v*_*k*_) ∈ *cs*(*H*), the sequence (*w*_*n*_) defined by *w*_*n*_ = ∑_*k*=0_^*n*^ *v*_*k*_ for all *k* ∈ *N*, is an element of the space *c*(*H*). Since {*c*(*H*)}^*α*^ = *l*_1_(*H*), the series ∑_*k*_ *w*_*k*_(*u*_*k*_ − *u*_*k*+1_) is convergent. Also, we have
  (54) ∑k=mnwk−wk−1uk≤∑k=mn−1wkuk−uk+1 +wnun−wm−1um.
 Since (*w*_*n*_) ∈ *c*(*H*) and (*u*_*k*_) ∈ *bv*(*H*) ⊂ *c*(*H*), the right-hand side of inequality (54) converges to zero as *m*, *n* → *∞*. Hence, the series ∑_*k*=0_^*∞*^(*w*_*k*_ − *w*_*k*−1_)*u*_*k*_ or ∑_*k*=0_^*∞*^ *u*_*k*_ *v*_*k*_ converges and *bv*(*H*)⊆{*cs*(*H*)}^*β*^.
(c) By using (a), it is known that *bv*(*H*)⊆{*cs*(*H*)}^*β*^ and since {*cs*(*H*)}^*β*^ ⊂ {*cs*(*H*)}^*γ*^, so *bv*(*H*)⊂{*cs*(*H*)}^*γ*^. We need to show that {*cs*(*H*)}^*γ*^ ⊂ *bv*(*H*). Let *u* = (*u*_*n*_)∈{*cs*(*H*)}^*γ*^ and *v* = (*v*_*n*_) ∈ *c*_0_(*H*). Then, for the sequence (*w*_*n*_) ∈ *cs*(*H*) defined by *w*_*n*_ = (*v*_*n*_ − *v*_*n*+1_) for all *n* ∈ *N*, we can find a number *K* > 0 such that $H^{s}(\sum_{k=0}^{n}u_{k}w_{k},\bar{0})\leq K$ for all *n* ∈ *N*. Since (*v*_*n*_) ∈ *c*_0_(*H*) and (*u*_*n*_)∈{*cs*(*H*)}^*γ*^ ⊂ *l*_*∞*_(*H*), there exists a real number *M* > 0 such that $H^{s}(u_{n}v_{n},\bar{0})\leq M$ for all *n* ∈ *N*. Therefore,
  (55) Hs∑k=0nuk−uk−1vk,0¯ ≤Hs(∑k=1n+1uk(vk−vk+1),0¯)  +Hs(vn+2un+1,0¯)≤K+M.
 Hence (*u*_*k*_ − *u*_*k*−1_)∈{*c*_0_(*H*)}^*γ*^ = {*c*_0_(*H*)}^*α*^ = *l*_1_(*H*); that is, (*u*_*n*_) ∈ *bv*(*H*). Therefore, since the inclusion {*cs*(*H*)}^*γ*^ ⊂ *bv*(*H*) holds, we conclude that {*cs*(*H*)}^*γ*^ = *bv*(*H*), as desired.

## 5. Conclusion

Partial metrics are more flexible than metrics; they generate partial orders and their topological properties are more general than the one for metrics, argued by the fact that the self-distance of each point need not be zero. They are useful in partially defined information for the study of domains and semantics in computer science.

The concept of level sets associated with a fuzzy set was originally introduced by Zadeh. With the aid of level sets we are able to provide a formulation for a fuzzy set in terms of crisp subsets via the representation theorem. The importance of having such a representation is that it can allow us to extend operations defined on crisp sets to the case of fuzzy sets. Our focus here is on using the idea of level sets to construct the sets of sequences of fuzzy numbers within partial metric spaces.

This work presents the alpha-, beta-, and gamma-duals of the sets of bounded, convergent, and null series and the set of sequences of bounded variation of fuzzy level sets, based on the partial metric. The potential applications of the obtained results include the characterization of matrix transformations between these sets of sequences.
